# Gene Therapy Knowledge and Attitude Among Healthcare Professionals: A Cross-Sectional Study

**DOI:** 10.3389/fpubh.2021.773175

**Published:** 2021-11-15

**Authors:** Maha M. AlRasheed, Hatoon AlAli, Abdulrahman F. Alsuwaid, Suhail Khalaf, Sondus I. Ata, Nasser F. BinDhim, Dana Bakheet, Fowad Khurshid, Tariq M. Alhawassi

**Affiliations:** ^1^Department of Clinical Pharmacy, College of Pharmacy, King Saud University, Riyadh, Saudi Arabia; ^2^Department of Clinical Laboratory Sciences, College of Applied Medical Sciences, King Saud University, Riyadh, Saudi Arabia; ^3^Pharmacy Services, King Saud University Medical City, Riyadh, Saudi Arabia; ^4^Sharik Association for Health Research, Riyadh, Saudi Arabia; ^5^Saudi Food and Drug Authority, Riyadh, Saudi Arabia; ^6^College of Medicine, Alfaisal University, Riyadh, Saudi Arabia; ^7^Center for Genomic Medicine, Research Center, King Faisal Specialist Hospital and Research Center, Riyadh, Saudi Arabia; ^8^Medication Safety Research Chair, College of Pharmacy, King Saud University, Riyadh, Saudi Arabia

**Keywords:** gene therapy, healthcare professionals, knowledge, attitude, Saudi Arabia

## Abstract

This study aimed to assess healthcare professionals' knowledge, attitudes, and concerns toward gene therapy in Saudi Arabia. We conducted an online cross-sectional survey via convenience sampling during the period from December 2018 to March 2019. A total of 419 (358 pharmacists and 61 physicians) responded to our questionnaire. Three hundred and nine (73.7%) were male, and the mean (±SD) age of 32.0 ± 7.7 years. The mean knowledge scores of all participants, pharmacists, and physicians were 3.8 ± 1.9, 3.8 ± 1.9, and 3.7 ± 1.9, respectively (*P* = 0.73). Higher knowledge score was associated with younger age (Coefficient: −0.03; *P* = 0.02), male (Coefficient: 0.57; *P* = 0.01), master's degree (Coefficient: 0.93; *P* = 0.003) and Ph.D. holders (Coefficient: 1.10; *P* = 0.01), and participants graduated from Canada (Coefficient: 2.10; *P* = 0.01).Moreover, about half of the respondents (55%) were concerned about gene therapy, and genetics training at college was considered the best gene therapy education method by 69.4%. Attitude score was not significantly associated with the profession (*P* = 0.88) but positively correlated with the knowledge score (rho= 0.4; *P* < 0.001). In conclusion, pharmacists and physicians showed limited knowledge with a positive attitude toward gene therapy. Therefore, educational programs on gene therapy need to be considered, focusing primarily on the safety, and social acceptance of such new therapeutic management.

## Introduction

Gene therapy is a medical procedure in which a healthy genetic material is introduced into a patient's cells to replace defective genetic material (1). More than twenty gene and cell-based gene therapy products have already been approved (2). A similar number or even more new gene therapy drugs in the pipeline are anticipated to be approved by regulatory bodies in the next 5 years (3), with a significant upswing in the investments in such technologies.

Driven by the positive and evident impact as well as clinical and economic outcomes, gene therapy is emerging as a method that potentially offers new and unique approaches to treating devastating, rare, and inherited diseases as well as incurable illnesses or those with limited alternative therapies (4, 5). However, the process remains complex. The currently used techniques need better understanding by health care professionals (HCPs), entailing comprehensive grasping of the ethical issues that cover the use of this procedure (6). For these reasons, there is a need to address healthcare providers' knowledge, perceptions, and confidence toward gene therapy and its implementation and tailor such research further for the local context.

Although numerous studies on this subject have been carried out among clinicians and the general public, they have mostly been confined to developed countries (7–9). For example, a study assessing the knowledge and attitudes of medical residents in Rome concluded that Italian residents have insufficient knowledge on genetic testing for colorectal cancer (7). In the United States, a study evaluating U.S. public health educators' attitudes toward genomic competencies, their awareness, and their basic and applied genomic knowledge revealed unfavorable attitudes and limited genomic knowledge among this group of HCPs (8). Besides, a Chinese study highlights the lack of knowledge on gene therapy among many public and around one-third of clinicians in China (9). Hitherto, little is known about similar outcomes in the Eastern Mediterranean region despite that genetic disorders are not uncommon in the region due to several factors, including consanguinity (10). Only two studies have been published in the region, one in Qatar (11) and another in Kuwait (12) to date. Practicing physicians and pharmacists in Saudi Arabia have diverse educational backgrounds, having trained in Saudi Arabia, the United States of America, Canada, the United Kingdom, India, and many other Middle Eastern, European, and Asian medical schools (13). However, no published studies are available on the current status of the knowledge and attitudes toward gene therapy among HCPs in Saudi Arabia. Consequently, a cross-sectional survey of physicians and pharmacists working in Saudi Arabia aimed to understand their knowledge, attitudes, and concerns related to therapeutic modalities.

## Methodology

### Study Design and Setting

A cross-sectional survey was conducted via convenience sampling during the period from December 2018 to March 2019. Eligible participants included practicing physicians and pharmacists working in different healthcare institutions and hospitals. The study sample size was based on the assumption that the proportion of responses to most of the main questions would be about 70%. No previous studies are available on this subject from Saudi Arabia. The survey was distributed through WhatsApp as a snowball method with an expectation to reach 2,000 participants, and the expected good gene knowledge was 75% among the participants. The estimated sample required was 360 participants with a margin of error of ± 0.05 and a confidence level of 95%.

Ethical approval for this study was obtained from King Saud University College of Medicine Institutional Review Board with approval number E-18-3495.

### Study Population

A cohort of physicians and pharmacists was randomly selected and first contacted electronically and provided an explanation of the study's objectives to guarantee a good response rate and participation. Those who agreed to participate were then offered the questionnaires. The study survey was made available on the online survey platform “Google Forms,” which is considered user-friendly and easily accessible with the different web browsers (14). Only one reminder was sent to participants 2 weeks later. The participants were assured of personal information confidentiality and asked to complete the written consent before contributing to the study.

### Study Questionnaire

Based on an extensive literature review of previously published studies, the survey was refined from validated questionnaires that were previously used to address our objectives (11, 12). The research group established the content validity of the adapted questionnaire at the Department of Clinical Pharmacy, King Saud University, with extensive knowledge of the study field. The face validity of the survey was assessed by ten expert HCPs (five pharmacists and five physicians) to assure the clarity and the premise of each question within the questionnaire. The survey was amended after reviewing the received feedback. The survey was then piloted for content, design, readability, and comprehension by another ten HCPs (five pharmacists and five physicians). Suitable amendments were made based on their feedback to develop the final questionnaire. The self-reported questionnaire comprised a series of questions to assess respondents: (i) demographics and personal and professional characteristics; (ii) their perceived level of knowledge (*n* = 9) and attitudes toward gene therapy and its application (*n* = 4); (iii) their self-estimated level of knowledge and training needs (*n* = 4); (iv) reasons to be concerned and not to be concerned about gene therapy (*n* = 2). A list of reasons was provided, and participants were allowed to choose one or more if they wished. Knowledge was assessed by giving 1 to the correct answer and 0 to the wrong answer. The scale measured knowledge of a maximum of 9 to minimum 0. A score of <4 was taken as poor while ≥ 4 as good. The attitude score consisted of four items; participants were given 1 if they answered yes and 0 for other answers.

### Statistical Analysis and Data Presentation

Continuous variables were checked for normality using the Shapiro-Wilk test, and normally distributed variables were compared using the student *t*-test, and non-normally distributed variables were compared using the Mann-Whitney test. Homogeneity of variances was checked using Levene's test before the *t*-test. Continuous data were described as mean and standard deviation, and categorical variables as frequencies and percentages. Categorical data were compared using the Chi-square test or Fisher exact test if the expected frequency was <5. Knowledge and attitude scores were calculated by giving 1 point for correct answers and 0 points for wrong or “I don't know” answers. Cronbach's alphas were calculated to estimate the internal reliability of items relating to respondents about gene therapy using the entire sample of participants (reliability coefficient = 0.77). Correlation between knowledge and attitude scores was assessed using the Spearman correlation. Stata 16.1 was used to perform the statistical analyses (Stata Corp- College Station- TX- USA), and a P-value of less the 0.05 was considered statistically significant.

### Regression Analysis

Linear regression analysis was used to assess factors affecting the knowledge score (normally distributed). Collinearity was tested for factors included in Table 1 with variance inflation factor (VIF), and the “years of experience” was omitted. All variables included in the final model had a VIF of <1.3.

**Table 1:** Socio-demographic characteristics of the study participants.

**Variables**	**Physicians**	**Pharmacists**	**Total**	***P***
	**(*n* = 61)**	**(*n* = 358)**	**(*n* = 419)**	
Male	37 (61%)	272 (76%)	309 (73.7%)	0.01
Age (Years)	36.1± 10.4	31.3± 7.0	32.0± 7.7	<0.001
**Educational level**				
Bachelor's degree	15 (24.6%)	261 (72.9%)	276 (65.9%)	<0.001
Master's degree	6 (9.8%)	41 (11.4%)	47 (11.2%)	
Ph.D.	5 (8.2%)	39 (10.9%)	44 (10.5%)	
Residency	16 (26.2%)	13 (3.6%)	29 (6.9%)	
Fellowship	17 (27.9%)	4 (1.1%)	21 (5%)	
MD	1 (1.6%)	0	1 (0.2%)	
Other^∧^	1 (1.6%)	0	1 (0.2%)	
**Position**				
Academic	8 (13.1%)	43 (12%)	51 (12.2%)	0.04
Clinical	48 (78.7%)	234 (65.4%)	282 (67.3%)	
Administrative	5 (8.2%)	81 (22.6%)	86 (20.5%)	
**Experience in years**				
<5	22 (36.1%)	144 (40.2%)	166 (39.6%)	0.046
5–10	15 (24.6%)	130 (36.3%)	145 (34.6%)	
11–20	17 (27.7%)	65 (18.2%)	82 (19.6%)	
More than 20	7 (11.5%)	19 (5.3%)	26 (6.2%)	
**Country of graduation**				
Saudi Arabia	42 (68.8%)	189 (52.8%)	231 (55.1%)	<0.001
USA	1 (1.6%)	15 (4.2%)	16 (3.8%)	
UK	2 (3.3%)	13 (3.6%)	15 (3.6%)	
Canada	4 (6.5%)	2 (0.5%)	6 (1.4%)	
Australia	1 (1.6%)	1 (0.3%)	2 (0.5%)	
Other ^#^	11 (18%)	138 (38.5%)	149 (35.6%)	
**Time spent in clinical practice**				
<25%	5 (8.2)	73 (20.2)	78 (18.6)	<0.001
25–50%	9 (14.7)	47 (13.1)	56 (13.4)	
>50%	16 (26.2)	39 (10.9)	55 (13.1)	
100%	24 (39.3)	26 (7.3)	50 (11.9)	
Not working in clinical practice	7 (11.5)	173 (48.3)	180 (42.9)	

∧ *Other levels of education are specialized training programs/examination (e.g., MRCPCH)*.

# *Other countries include Egypt, Sudan, Jordan, Japan, Syria, Pakistan, Kuwait, Yemen, Germany, India, Bahrain, Sweden, and Iraq*.

*Continuous data are presented as mean and S.D., and categorical data as number and percentage*.

Normality of the residuals was tested with the Shapiro-Wilk test, and Breusch-Pagan was used to test for heteroskedasticity (*P* = 0.75). Link test was used to test model specification (Predicted value (_hat) *P* = 0.01, linear predicted value squared (_hatsq) *P* = 0.41), and a regression specification error test was used for omitted variables (*P* = 0.26). These results indicate proper model specification. Poisson regression was used to assess factors associated with the attitude score. The relation between knowledge and attitude scores was assumed to be non-linear, and locally weighted scatterplot smoothing (LOWESS) was used to plot the relationship between the two scores.

## Results

### Socio-Demographic Characteristics

A total of 419 healthcare professionals (HCPs) participated in the study. Of these, 358 (85.4%) were pharmacists, and 61 (14.6%) were physicians. Of the respondents, 309 (73.7%) were male with a mean (±SD) age of 32.0 ± 7.7 years. About two-thirds of the respondents (65.9%) were Bachelor's degree holders. Respondents' demographics characteristics and professional information are summarized in Table 1.

### Assessment of General Knowledge of Gene Therapy

In the second section of the survey, we explored HCP's knowledge about gene therapy. The mean knowledge scores of all participants, pharmacists, and physicians were 3.8 ± 1.9, 3.8 ± 1.9, and 3.7 ± 1.9, respectively. There was no statistically significant difference in knowledge between pharmacists and physicians (*P* = 0.73).

Most of the respondents (*n* = 366; 87.4%) knew gene therapy as an experimental technique that uses genes to treat or prevent disease(s). In addition, 27.7% of the participants thought that gene therapy is currently available in a research setting only. However, more than half (*n* = 218; 52.0%) of the participants were aware that gene therapy could be targeted to egg and sperm cells allowing the inserted gene to be passed on to future generations. Moreover, less than half of the respondents (*n* = 190; 45.3%) thought that the procedure could have very serious health risks, such as toxicity, inflammation, and cancer (Table 2).

**Table 2:** Information about the respondent's knowledge of gene therapy.

**Statement**	**Physicians**	**Pharmacists**	**Total**	***P***
	**(*n* = 61)**	**(*n* = 358)**	**(*n* = 419)**	
Gene therapy is an experimental technique that uses genes to treat or prevent disease(s)	49 (80.3%)	317 (88.5%)	366 (87.4%)	0.16
The U.S. Food and Drug Administration (FDA) has approved only a limited number of gene therapy products for sale in the United States	4 (6.6%)	34 (9.5%)	38 (9.1%)	0.63
Gene therapy is currently available in a research setting only	16 (26.2%)	100 (27.9%)	116 (27.7%)	0.67
Gene therapy can have very serious health risks, such as toxicity, inflammation, and cancer	23 (37.7%)	167 (46.7%)	190 (45.3%)	0.15
Gene therapy could be targeted to egg and sperm cells which would allow the inserted gene to be passed to future generations	24 (39.3%)	194 (54.2%)	218 (52.0%)	0.06
Gene therapy is approved only for adults	26 (42.6%)	123 (34.4%)	149 (35.5%)	0.64
How do you think gene therapy works?	36 (59%)	221 (61.7%)	257 (61.3%)	0.38
Which cells do you think are targeted by gene therapy?	22 (36.1%)	96 (26.8%)	118 (28.2%)	0.05
What type of vector is used to carry modified genes in the targeted cells?	3 (4.9%)	15 (4.2%)	18 (4.3%)	0.73

*Knowledge was assessed by giving 1 to the correct answer and 0 to the wrong answer. The scale measured knowledge of a maximum of 9 to minimum 0. A score of <4 was taken as poor while ≥ 4 as good*.

*Data are presented as numbers and percentages. Numbers presented refer to the number of participants who answered the questions correctly*.

Additionally, 54% of the pharmacists, compared to 39% of the physicians, correctly answered the statement, “Gene therapy could be targeted to egg and sperm cells which would allow the inserted gene to be passed to future generations.” Similarly, 73.2% of the participating pharmacists and 63.9% of the physicians did not know which cells are targeted by gene therapy (Table 2).

### Self-Assessed Knowledge of Gene Therapy

Table 3 reports the self-assessed level of knowledge and suggested approaches to educate participants on gene therapy. A clear majority of the respondents (*n* = 349; 83.3%) were aware of the meaning of gene therapy, while only a smaller proportion (*n* = 120; 28.6%) reported being well-informed about it in detail (Table 3).

**Table 3:** Self-assessed knowledge and suggested approaches to educate gene therapy.

**Statement**	**Physicians**	**Pharmacists**	**Total**	***P***
	**(*n* = 61)**	**(*n* = 358)**	**(*n* = 419)**	
**Do you know the meaning of gene therapy?**				
Yes No I do not know	50 (82%) 6 (9.8%) 5 (8.2%)	299 (83.5%) 32 (8.9%) 27 (7.5%)	349 (83.3%) 38 (9.1%) 32 (7.6%)	0.78
**Overall, I feel that I am well-informed about gene therapy**				
Yes No I do not know	7 (11.5%) 39 (63.9%) 15 (24.6%)	113 (31.6%) 173 (48.3%) 72 (20.1%)	120 (28.6%) 212 (50.6%) 87 (20.8%)	0.01
From where you have learned about gene therapy^**^				
School Practice Seminar Media Journal Colleagues	20 (32.8%) 27 (44.3%) 19 (31.2%) 34 (55.7%) 26 (42.6%) 26 (42.6%)	108 (30.2%) 78 (21.8%) 56 (15.6%) 131 (36.6%) 80 (8.7%) 135 (37.7%)	128 (30.5%) 105 (25.1%) 75 (17.9%) 165 (39.4%) 106 (25.3%) 161 (38.4%)	0.001
The best way to educate HCPs about gene therapy^**^				
During college studies	37 (60.7%)	254 (70.9%)	291 (69.4%)	0.02
During residency training	34 (55.7%)	168 (46.9%)	202 (48.2%)	
Seminars	30 (49.2%)	174 (48.6%)	204 (48.7%)	
Continuous medical education (CME)	38 (62.3%)	152 (42.5%)	190 (45.3%)	
Scientific journals	28 (45.9%)	135 (37.7%)	163 (38.9%)	
Grand rounds	18 (29.5%)	100 (27.9%)	118 (28.2%)	

** *Multiple responses*.

*Data are presented as numbers and percentages*.

### Gene Therapy Education

The largest proportion of respondents indicated that they have learned about gene therapy from the media (*n* = 165, 39.4%), followed by colleges (*n* = 161, 38.4%). In addition, the majority of them felt that the best way to educate HCPs about gene therapy was during college studies (*n* = 291, 69.4%) or through seminars (*n* = 204, 48.7%), or during residency training (*n* = 202, 48.2%) (Table 3).

### Relationship Between Socio-Demographic Factors and Gene Therapy Knowledge

High knowledge score was associated with younger age, male gender, Master's and Ph.D. degree holders, graduation from Canada or other countries (Table 4). Knowledge score was not associated with the profession.

**Table 4:** Linear regression analysis for factors affecting knowledge score (*n* = 419).

	**Coefficient (95% confidence interval)**	***P***
Age	−0.03 (−0.06 to −0.005)	0.02
Male	0.57 (0.13 to −1.01)	0.01
Profession	0.17 (−0.45 to 0.78)	0.60
**Educational level**		
Master vs. Bachelor's Ph. D. vs. Bachelor's	0.93 (0.33 to 1.53) 1.10 (0.34 to 1.86)	0.003 0.01
**Country of graduation**		
Canada vs. Saudi Arabia Others vs. Saudi Arabia	2.10 (0.57 to 3.60) 0.63 (0.21 to 1.05)	0.01 0.003
Position	−0.20 (−0.55 to 0.14)	0.25
Not working in clinical setting	−0.12 (−0.23 to −0.01)	0.04

### General Attitudes Toward Gene Therapy

Respondents' attitudes toward gene therapy are reported in Table 5. When HCPs were asked whether they believed that gene therapy is or will soon become a useful treatment strategy, 81.4% (*n* = 341) said that they would be, while 3.8% (*n* = 16) said that they would not. The remaining 14.8% (*n* = 62) indicated that they did not know (Table 5).

**Table 5:** Information about the respondent's attitude toward gene therapy.

**Statement**	**Physicians**	**Pharmacists**	**Total**	***P***
	**(*n* = 61)**	**(*n* = 358)**	**(*n* = 419)**	
**I believe that gene therapy is or will soon become a useful treatment strategy**				
Yes No I do not know	47 (77.5%) 2 (3.3%) 12 (19.7%)	294 (82.1%) 14 (3.9%) 50 (14%)	341 (81.4%) 16 (3.8%) 62 (14.8)	0.49
**If it is possible to cure adults with debilitating diseases using gene therapy, do you agree that those people ought to be allowed to be treated by gene therapy?**				
Yes No I do not know	37 (60.7%) 7 (11.5%) 17 (27.9%)	219 (61.1%) 31 (8.7%) 108 (30.2%)	256 (61.1%) 38 (9.1%) 125 (29.8%)	0.76
**If it is possible to cure children with the usually fatal genetic disease, do you agree that those children ought to be allowed to be treated using gene therapy?**				
Yes No I do not know	47 (77%) 3 (5%) 11 (18%)	213 (59.5%) 43 (12%) 102 (28.5%)	260 (62%) 46 (11%) 113 (27%)	0.03
**Are you concerned about the use of gene therapy?**				
Yes No	35 (57.4%) 26 (42.6%)	194 (54.2%) 164 (45.8%)	229 (54.7%) 190 (45.3%)	0.64

*Data are presented as numbers and percentages*.

Moreover, when the study cohort was asked whether they agreed with the statement, “If it is possible to cure adults with debilitating diseases (e.g., Alzheimer or Parkinson disease) and children with usually fatal genetic disease (e.g., sickle cell anemia, muscular dystrophy, etc.) using gene therapy,” more than 61% (*n* = 256) of the respondents agreed to gene therapy being allowed to be used in such populations (Table 5).

### Concerns About Gene Therapy

More than half (54.7%, *n* = 229) of respondents agreed when asked if they were concerned about the use of gene therapy. The most frequent reasons for the concern were patient safety (30.3%, *n* = 127), high cost (26.5%, *n* = 111), adverse events (22.7%, *n* = 95), and a belief that genetic changes would be passed on to offspring (21.0%, *n* = 88).

While the greatest reason for not being concerned about gene therapy was that it would only be prescribed for particular conditions (fatal conditions) and certain patients (20.5%, *n* = 86), the procedure would be well-regulated and not go against nature (20.0%, *n* = 84), and it was a product of high technology; therefore, safety should not be a concern (15.5%, *n* = 65).

### The Attitude Toward Gene Therapy

**Figure 1:**
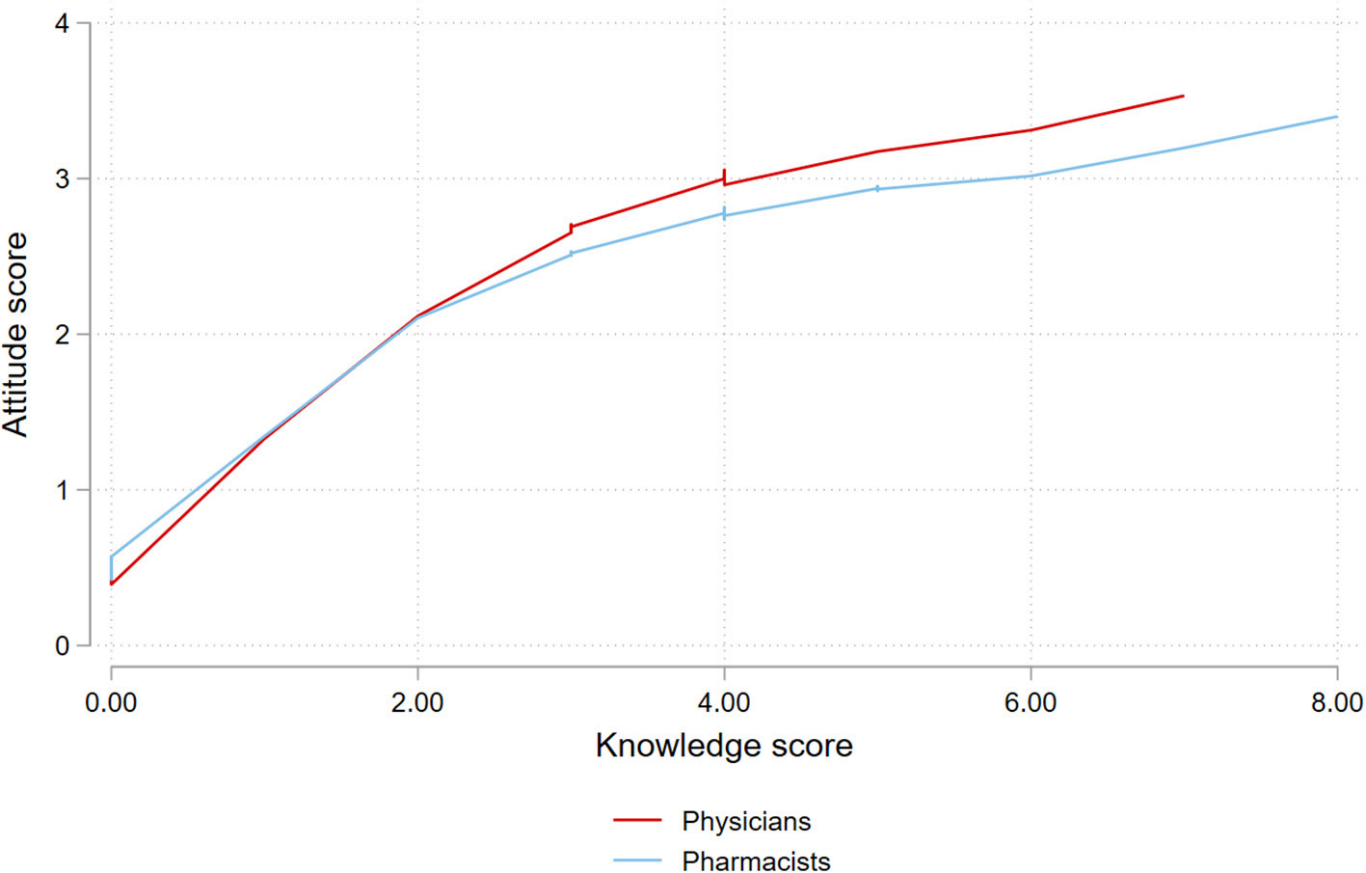
Locally weighted scatterplot smoothing (LOWESS) of the attitude and knowledge scores.

The mean attitude score was 2.7 ± 1.3 among physicians and 2.6 ± 1.3 among pharmacists (*P* = 0.35). Attitude score was not significantly associated with the profession (*P* = 0.88). However, there was a positive correlation between knowledge and attitude (rho 0.4, *P* < 0.001). The relation between both scores is plotted in Figure 1.

## Discussion

This is the first survey that aimed to explore the knowledge and attitude toward gene therapy among HCPs in Saudi Arabia to the best of our knowledge. Gene therapy has been an acquainted theme in the medical research community for the past few years (15). However, one of the main challenges has been the lack of familiarity and understanding of this technique among HCPs. The study revealed low awareness and limited knowledge about gene therapy among the participating physicians and pharmacists. Furthermore, most respondents regarded it as essential to improve the understanding of the procedure and considered college studies, seminars, or residency training as the best way to educate HCPs. In Kuwait, physicians and pharmacists revealed little awareness regarding pharmacogenetics and pharmacogenetics testing, and 16.0% showed confidence in applying these tests in their practice settings (12). In addition, another cross-sectional survey comparing the level of awareness and attitude toward pharmacogenomics of pharmacists vs. doctors within a large medical corporation in Qatar reported a low level of awareness toward pharmacogenomics among participants (11). Besides, a study conducted in Malaysia found that most respondents had poor to fair knowledge, and nearly half had no pharmacogenomics education (16). These results point to the need for better strategies and guidelines for enlightening HCPs on gene therapy. Recent research demonstrated a positive impact on improving physicians' knowledge and confidence in using genetic services resulting from availing those genetics educational outreaches (17, 18). Our study highlights the need to develop strategies to ensure multi-faceted, accessible educational outreach programs for the medical community at large in Saudi Arabia. Such programs are essential to correct any misconceptions related to gene therapy and will facilitate future implications of gene therapy in different diseases (19). Moreover, several countries had launched gene therapy educational programs to both healthcare professionals and patients (20).

Rather than addressing disease symptoms, gene therapy can address the core causes of genetic illnesses by changing the expression of a patient's genes or repairing or replacing a defective gene (21). Three gene therapies have been approved for human medical use in the United States in 2017 (22–24), with many more approvals being expected to follow in the near future. The results from this study provide acumens into HCP attitudes toward gene therapy. Our findings indicated that most participants accepted the technique for conditions perceived as severe and fatal genetic diseases. Furthermore, both clinicians and pharmacists believe that the technique will soon become a useful treatment strategy and would be beneficial for improving human health in the near future. Hence, HCPs in this study expressed a positive attitude and a desire to adopt the strategy into their practices in the near future.

Today, gene therapy holds promise for treating a wide range of genetic and non-genetic diseases, such as cancer (25, 26), cystic fibrosis (27, 28), heart disease (29, 30), and diabetes (31, 32). Moreover, the participants in this study strongly supported gene therapy to treat fatal or debilitating diseases in both adults and children. Similarly, Wang et al. (9) reported a broader acceptance of gene therapy to treat fatal diseases by both clinicians and the public (83 and 88%, respectively). On the other hand, Xiang et al. (33) found a large proportion of respondents not accepting gene therapy for complex and potentially severe diseases such as breast cancer (63.7%) and congenital heart disease (60.3%).

Gene therapy involves changing the body's genetic setup, raising many unique medical and ethical concerns. Many people oppose gene therapy on religious grounds, believing that altering genetic material is against God's will (34). In recent years, the widely reported occurrence of adverse events in gene therapy clinical trials had strengthened the fear in public perceptions of the therapeutic approach (35). Indeed, medical concerns, including patient safety, high cost and adverse events, and ethical issues, are the main concerns raised by the participants in our study. This trend has also been described in other studies (9, 33). A study investigating current opinions of clinical genetic professionals on genome editing reported concerns about the safety and ethical aspects of the technology, as well as fears over its potentially inappropriate applications (36).

One of the limitations of this study relates mainly to self-report by participants as the survey relied on their conviction to appraise their knowledge and attitude. Thus, some participants may have overestimated or underestimated their capabilities in responding to the questions, possibly leading to recall bias. Also, the cross-sectional nature of the survey represented one point in time, limiting the ability to generalize this finding to all healthcare providers in Saudi Arabia. The study included physicians and pharmacists only since they are the first-line healthcare workers dealing with gene therapy. Future studies including all healthcare professionals are recommended. Moreover, there is an imbalance in gender distribution among participants; however, this distribution reflects the gender distribution among healthcare professionals in Saudi Arabia (37).

## Conclusion

Our findings highlight limited knowledge but a positive attitude toward gene therapy among HCPs in Saudi Arabia. The safety of gene therapy was among the primary concerns for both clinicians and pharmacists. Our results point to a need for both clinicians and pharmacists to be more aware of the progress in gene therapy and its implications. Educational programs about the procedure need to be considered and should focus on the safety and social acceptance of such new therapeutic management.

## Data Availability Statement

The raw data supporting the conclusions of this article will be made available by the authors, without undue reservation.

## Ethics Statement

The studies involving human participants were reviewed and approved by King Saud University College of Medicine Institutional Review Board (Approval No. E-18-3495). The patients/participants provided their written informed consent to participate in this study.

## Author Contributions

MA, TA, SA, HA, and FK conceptualized the study. MA, AA, SK, and NB collected data. MA analyzed and interpreted the data. MA and FK wrote the first draft. MA, TA, SA, DB, and FK reviewed and edited for the final manuscript. All authors approved the final version.

## Conflict of Interest

The authors declare that the research was conducted in the absence of any commercial or financial relationships that could be construed as a potential conflict of interest.

## Publisher's Note

All claims expressed in this article are solely those of the authors and do not necessarily represent those of their affiliated organizations, or those of the publisher, the editors and the reviewers. Any product that may be evaluated in this article, or claim that may be made by its manufacturer, is not guaranteed or endorsed by the publisher.
